# Supplementary material for: Cortactin stabilizes actin branches by bridging activated Arp2/3 to its nucleated actin filament
Source: Nat Struct Mol Biol. 2024 Jan 24;31(5):801–9. doi: 10.1038/s41594-023-01205-2 (PMC11102864; doi:10.1038/s41594-023-01205-2)
Supplement: Supplementary file 1 — Reporting Summary [file 41594_2023_1205_MOESM1_ESM.pdf]

nature portfolio

Corresponding author(s): .Carolyn Moores; Michael Way

Last updated by author(s): 2023-12-11

Reporting Summary

Nature Portfolio wishes to improve the reproducibility of the work that we publish. This form provides structure for consistency and transparency in reporting. For further information on Nature Portfolio policies, see our Editorial Policies and the Editorial Policy Checklist.

Statistics

For all statistical analyses, confirm that the following items are present in the figure legend, table legend, main text, or Methods section.

n/a	Confirmed	
☐	☒	The exact sample size (*n*) for each experimental group/condition, given as a discrete number and unit of measurement
☐	☒	A statement on whether measurements were taken from distinct samples or whether the same sample was measured repeatedly
☐	☒	The statistical test(s) used AND whether they are one- or two-sided<br>*Only common tests should be described solely by name; describe more complex techniques in the Methods section.*
☒	☐	A description of all covariates tested
☒	☐	A description of any assumptions or corrections, such as tests of normality and adjustment for multiple comparisons
☐	☒	A full description of the statistical parameters including central tendency (e.g. means) or other basic estimates (e.g. regression coefficient) AND variation (e.g. standard deviation) or associated estimates of uncertainty (e.g. confidence intervals)
☐	☒	For null hypothesis testing, the test statistic (e.g. *F*, *t*, *r*) with confidence intervals, effect sizes, degrees of freedom and *P* value noted<br>*Give P values as exact values whenever suitable.*
☒	☐	For Bayesian analysis, information on the choice of priors and Markov chain Monte Carlo settings
☒	☐	For hierarchical and complex designs, identification of the appropriate level for tests and full reporting of outcomes
☒	☐	Estimates of effect sizes (e.g. Cohen's *d*, Pearson's *r*), indicating how they were calculated

Our web collection on statistics for biologists contains articles on many of the points above.

Software and code

Policy information about availability of computer code

Data collection	Cryo-EM dataset of cortactin stabilized actin branch junction was collected on Titan Krios with EPU software (version 2.14, Thermo Fisher Scientific). The data were collected with a K3 detector operating in super-resolution mode (bin2) with a BioQuantum energy filter (Gatan).The debranching essays were performed with TIRF microscopy (Nikon TiE inverted microscope, iLAS2, Gataca Systems) equipped with a 60× oil-immersion objective. Images were acquired using an Evolve EMCCD camera (Photometrics), controlled with the Metamorph software (version 7.10.4, from Molecular Devices).
Data analysis	Cryo-EM data was processed using CryoSPARC v3.Model was built using AlphaFold Monomer v2.0 pipeline,ISOLDE,Namdinator and Coot. Rise and twist angles of actin and actin related proteins were calculated in PyMOL Molecular Graphics System, Version 2.5.4 Schrödinger, LLC. The distance between interacting atoms were measured in ChimeraX. Structural figures and movies were made with ChimeraX. The debranching rate was analyzed with Fiji (version 2.14.0/1.54f).

For manuscripts utilizing custom algorithms or software that are central to the research but not yet described in published literature, software must be made available to editors and reviewers. We strongly encourage code deposition in a community repository (e.g. GitHub). See the Nature Portfolio guidelines for submitting code & software for further information.

Data

Policy information about availability of data

All manuscripts must include a data availability statement. This statement should provide the following information, where applicable:

- Accession codes, unique identifiers, or web links for publicly available datasets
- A description of any restrictions on data availability
- For clinical datasets or third party data, please ensure that the statement adheres to our policy

The cryo-EM reconstructions are deposited in the Electron Microscopy Data Bank under the following accession codes: Daughter filament consensus reconstruction - EMDB-17553; Arp2/3 complex and cortactin locallyl refined reconstruction – EMDB-17554; Daughter filament and cortactin locally refined reconstruction – EMDB-17555; Capping protein locally refined reconstruction – EMDB-17556; Mother filament locally refined reconstruction – EMDB-17557; mother filament consensus reconstruction – EMDB-17558. The corresponding composite structural model is deposited in the Worldwide Protein Data Bank under the accession code PDB ID: 8P94. PDB models used for structure comparison and model building are PDB ID: 8E9B and PDB ID: 6UHC. Source data are provided with this paper.

Research involving human participants, their data, or biological material

Policy information about studies with human participants or human data. See also policy information about sex, gender (identity/presentation), and sexual orientation and race, ethnicity and racism.

Reporting on sex and gender	n/a
Reporting on race, ethnicity, or other socially relevant groupings	n/a
Population characteristics	n/a
Recruitment	n/a
Ethics oversight	n/a

Note that full information on the approval of the study protocol must also be provided in the manuscript.

Field-specific reporting

Please select the one below that is the best fit for your research. If you are not sure, read the appropriate sections before making your selection.

☒ Life sciences ☐ Behavioural & social sciences ☐ Ecological, evolutionary & environmental sciences

For a reference copy of the document with all sections, see nature.com/documents/nr-reporting-summary-flat.pdf

Life sciences study design

All studies must disclose on these points even when the disclosure is negative.

Sample size	Cryo-EM sample size (12,073 movies) was determined based on previous similar studies (Ding et al.2022, PMID 35622886; Shaaban et al. 2020, 32839613), by microscope availability and data quality. After manually curating collected movies, 8,518 micrographs with CTF fit resolution < 6.4 Å and total full-frame motion distance < 50 pixels were selected for further analysis. For debranching assays, > 40 branches were chosen randomly for each condition and according to previously published analysis (Cao et al, 2023, PMID 36939020), which produces a survival curve of the branches that can be fit with an exponential equation and which is determined by branch disassociation rate.DATA EXCLUSIONS: Cryo-EM images were selected in a non-biased manner using well defined criteria (CTF fit resolution, total full-frame motion distance etc). For debranching assays, actin branches which are obviously abnormal, for example, sticking to the cover-slip surface, were excluded. REPLICATION: For the cryo-EM dataset, similar images were obtained from 2-3 preliminary test dataset. Data for 3D reconstruction were collected from one grid. For debranching assays, each experiment was repeated three times independently and all replication attempts were successful. RANDOMIZATION: During cryoEM data processing, each dataset was randomly split in two for calculating gold-standard Fourier Shell Correlation (FSC). For debranching assays, all branches were generated under the same condition and subsequently exposed to different experimental variables. Other variables such as temperature and tension on the filaments were kept constant. Actin branches were randomly picked at time zero for analysis. BLINDING: Blinding is not a common practice in cryo-EM where the experience of the researcher on sample behaviour will benefit the efficiency and accuracy of data collection. During data processing, the data were mostly automatically processed using unbiased software. Given the complex and flexible nature of our sample, researchers need to select the targeted 2D and 3D classes in an unblinded way for further data processing. Rigorous checking for model bias is performed at multiple stages. For debranching assays, once the imaging field of view is chosen, the imaging took place automatically. The investigator cannot manipulate data acquisition based on the experimental condition.
Data exclusions	
Replication	
Randomization	
Blinding	

Reporting for specific materials, systems and methods

We require information from authors about some types of materials, experimental systems and methods used in many studies. Here, indicate whether each material, system or method listed is relevant to your study. If you are not sure if a list item applies to your research, read the appropriate section before selecting a response.

Materials & experimental systems

n/a	Involved in the study
☒	☐ Antibodies
☒	☐ Eukaryotic cell lines
☒	☐ Palaeontology and archaeology
☒	☐ Animals and other organisms
☒	☐ Clinical data
☒	☐ Dual use research of concern
☒	☐ Plants

Methods

n/a	Involved in the study
☒	☐ ChIP-seq
☒	☐ Flow cytometry
☒	☐ MRI-based neuroimaging
